# Type 1 Diabetes and Other Autoimmune Diseases—Epidemiology, Pathophysiology and Screening

**DOI:** 10.1002/edm2.70119

**Published:** 2025-11-29

**Authors:** George J. Kahaly, Thomas Forst, Monika Kellerer, Stefanie Lanzinger, René D. Rötzer, Matthias Schott, Petra‐Maria Schumm‐Draeger

**Affiliations:** ^1^ Department of Medicine I Johannes Gutenberg University (JGU) Medical Center Mainz Germany; ^2^ Clinical Research Services Mannheim GmbH Mannheim Germany; ^3^ Centre for Internal Medicine 1, Marienhospital Stuttgart Germany; ^4^ Institute of Epidemiology and Medical Biometry, Ulm University Ulm Germany; ^5^ German Centre for Diabetes Research (DZD) Munich Germany; ^6^ Sciarc GmbH Baierbrunn Germany; ^7^ Division for Specific Endocrinology, Medical Faculty University Hospital Düsseldorf Düsseldorf Germany; ^8^ Center Internal Medicine Fünf Höfe Munich Germany

**Keywords:** antibody screening, autoimmune diseases, autoimmune polyendocrinopathy, polyglandular autoimmune syndromes, type 1 diabetes

## Abstract

**Introduction:**

The interplay between type 1 diabetes (T1D) and concomitant autoimmune diseases (AID) is both clinically and scientifically relevant. In this review, we delineate the epidemiological, pathophysiological and practical aspects underlying polyautoimmunity with a focus on T1D.

**Method:**

A comprehensive review of literature on T1D and associated AID was conducted, with the aim of drawing informed conclusions relevant to clinical practice. It draws on a targeted PubMed search conducted March–May 2025, emphasising recent peer‐reviewed articles in English.

**Results:**

Epidemiological data consistently indicate that individuals with T1D exhibit a significantly increased prevalence of additional AID. Familial aggregation of discordant AID and the concept of polyglandular autoimmune syndromes (PAS) or autoimmune polyendocrinopathy highlight that multiple AID can cluster and occur in a sequential and overlapping fashion, with T1D frequently acting as either an early or a subsequent manifestation. Thereby, genetic susceptibility, environmental triggers and epigenetic factors are pivotal in the initiation and progression of autoimmunity. Clinically, the coexistence of T1D with other AID poses significant challenges in disease management, often necessitating adjustments in therapeutic regimens and careful monitoring to mitigate complications. Early detection via stratified autoantibody testing is important for timely intervention and improved long‐term outcomes.

**Conclusions:**

Accordingly, screening for T1D‐associated autoantibodies in individuals with a personal or family history of AIDs, and vice versa, should be implemented in clinical practice.

## Graphical abstract

Individuals with type 1 diabetes exhibit significantly increased prevalence of additional autoimmune diseases. Epidemiological, familial and mechanistic evidence indicates frequent polyautoimmunity and shared genetic and environmental drivers. Coexistence complicates clinical management, and autoantibody‐based detection often identifies preclinical disease.

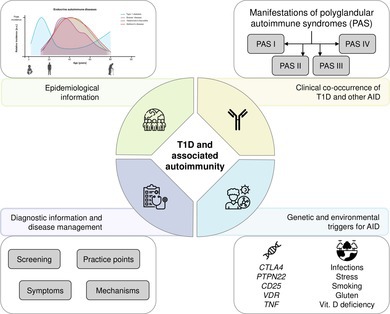

**Abbreviations**
17‐OH: 17‐alpha‐hydroxylase
21‐OH: 21‐hydroxylase
ACTH: adrenocorticotropic hormone
AD: Addison's disease
ADA: American Diabetes Association
AGA: American Gastroenterological Association
AID: autoimmune disease
AIRE: autoimmune regulator gene
AITD: autoimmune thyroid disease
AN: antinuclear
BACh2BTB: domain and CNC homologue 2
CCP: anti‐cyclic citrullinated peptide
CD: cluster of differentiation
CeD: celiac disease
CI: confidence interval
CRP: C‐reactive protein
CTLA4: cytotoxic T‐lymphocyte‐associated protein 4
DsDNA: double‐stranded DNA
ESR: erythrocyte sedimentation rate
ESsCD: European Society for the Study of Coeliac Disease
FT4: free thyroxine
GAD: glutamic acid decarboxylase
GD: Graves' disease
HbA1c: glycated haemoglobin
HLA: human leukocyte antigen
HT: Hashimoto's thyroiditis
IA‐2: tyrosine phosphatase‐related islet antigen 2
IC: islet cells
IF: intrinsic factor
IL2‐Rα: interleukin‐2 receptor α
LADA: latent autoimmune diabetes in adults
OGTT: oral glucose tolerance test
OR: odds ratio
PAS: polyglandular autoimmune syndrome
PC: parietal cells
POI: premature ovarian insufficiency
PTPN22: protein tyrosine phosphatase non‐receptor type 22
RA: rheumatoid arthritis
RBC: red blood cell
SIR: standardised incidence ratio
SLE: systemic lupus erythematosus
T1D: type 1 diabetes
Tg: thyroglobulin
TG: tissue transglutaminase
TNF: tumour necrosis factor
TPO: thyroid peroxidase
TSH: thyrotropin
VDR: vitamin D receptor
ZnT8: zinc transporter 8

## 1 Introduction

**FIGURE 1:**
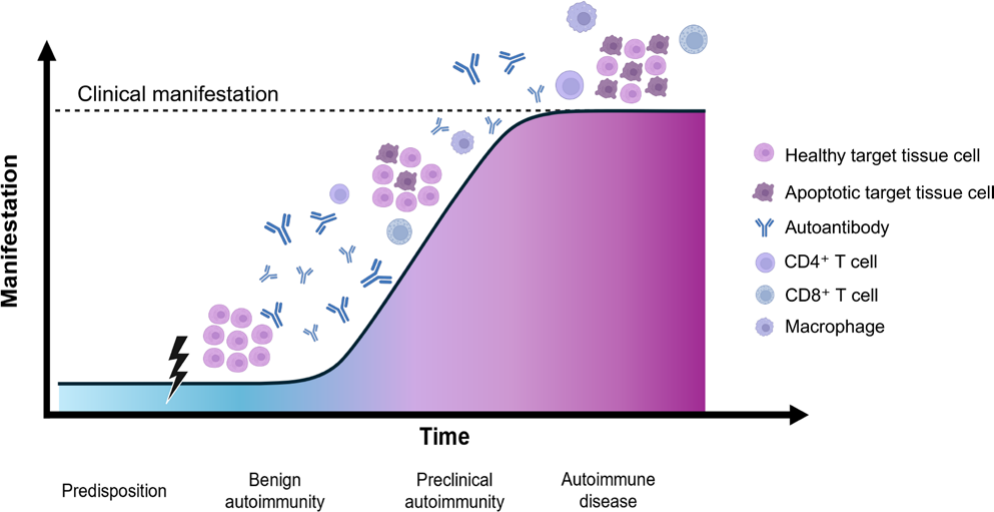
Development of autoimmune disease (AID). Genetically predisposed individuals may begin to develop an autoimmune response following an environmental trigger (flash). Autoantibodies serve as early indicators during the benign phase of autoimmunity. As the disease progresses, immune cells start to attack healthy target tissues, causing functional disturbances that can be detected in the preclinical stage. When the AID becomes clinically apparent, it can pose life‐threatening risks if left untreated.

Autoimmune diseases (AID) are defined by a self‐aggression of the body's own healthy structures through a dysfunctioning immune system. This can lead to either local or systemic chronic inflammatory processes, depending on whether the antigen is confined to specific cells or tissues or is expressed across various cell types. The underlying causes involve an interplay of genetic predispositions and environmental factors that trigger the onset of the diseases [1]. Clinical manifestations are often preceded by years of pathological processes characterised by autoantibody seroconversion (Figure 1) [2, 3, 4, 5]. Autoantibodies therefore serve as useful diagnostic markers, providing early indications of a potentially progressive disease [6]. Rarely do AID occur in the absence of detectable autoantibodies [7].

In type 1 diabetes (T1D), the dysregulated immune cells target pancreatic β cells. The disease is categorised into three stages: stage I is marked by the presence of beta cell autoimmunity (detection of at least two islet autoantibodies) with an unremarkable glucose metabolism; in stage II the autoimmunity is accompanied by dysglycemia; and stage III corresponds to clinical T1D due to pronounced autoimmune‐mediated insulin deficiency and corresponding hyperglycemia [8]. People living with T1D are prone to developing additional AIDs, and vice versa, which can significantly exacerbate the already substantial disease burden and complicate disease management.

The prevalence of T1D and other AID has shown a steady increase over recent decades and is anticipated to rise further [9, 10]. In 2022, T1D affected around 8.75 million people worldwide, with 1.52 million (17%) being under the age of 20. There were 530,000 new diagnoses of T1D that year, comprising 201,000 (38%) individuals younger than 20 years of age and 329,000 (62%) individuals aged 20 and above [11]. Regional incidence rates indeed confirm that more than half of new cases occur in adults [12]. This raises the question of what significance T1D holds as a primary and secondary manifestation in individuals with a history of autoimmunity, considering its frequent late onset.

## 2 Methods

This article provides a comprehensive review of the literature on T1D and its associated AID, with the aim of drawing informed conclusions relevant to clinical practice. Besides screening the relevant literature as stated below, the authors' extensive clinical and research experience was integrated in the manuscript to combine theoretical insights with practical considerations.

Relevant literature was identified through an iterative, targeted search of electronic databases, including PubMed, Cochrane Central, Medline, Scopus and Web of Science, conducted between March and May 2025. The search employed a combination of keywords and subject‐specific terminology related to T1D and associated AID. The Medical Subject Headings (MeSH) terms were the following: (1) (type 1 diabetes OR type I diabetes OR T1D) AND (other autoimmune disease OR polyglandular autoimmune syndrome OR multiple autoimmunity); (2) (type 1 diabetes OR type I diabetes OR T1D) AND (Autoimmune thyroid diseases OR Hashimoto OR Graves OR Celiac OR Crohn OR Autoimmune atrophic gastritis OR Vitiligo OR Rheumatoid arthritis OR Systemic lupus erythematosus OR Psoriasis OR Addison). Articles were included if they were published in English or German, focused on T1D and/or related autoimmune conditions, and presented either original data or substantial conceptual or theoretical contributions. Publications not directly related to the core themes of this review or written in languages other than English or German were excluded. Priority was given to recent, peer‐reviewed primary studies, population cohorts and high‐quality systematic reviews and meta‐analyses as well as clinical guidelines. Titles and abstracts were screened for relevance, and full texts of potentially eligible work were reviewed in detail for inclusion. The final selection and narrative synthesis were conducted by the co‐authors from relevant disciplines to reduce subjective bias. Because the aim was to provide an integrative narrative synthesis with expert clinical opinion, formal risk‐of‐bias assessment, systematic quality appraisal and exhaustive article counting were not undertaken.

## 3 Results

### 3.1 Associations Between Type 1 Diabetes and Other Autoimmune Diseases

#### 3.1.1 Epidemiological Insights

Several glandular and non‐glandular AID are associated with T1D. Table 1 lists epidemiological data for selected AID within the general population. A retrospective cohort study of 151 people with T1D found that 41 individuals (27.1%) had at least one additional AID, with diagnosis of the associated AID in 25 of the 41 affected individuals (60%) [31].

**TABLE 1:** Global and European epidemiological data for the general population.

Autoimmune disease		Estimate of the global prevalence	Estimate of the European prevalence	Estimate for sex predominance	
Type 1 diabetes		0.100% [11]	0.500%	Male	1.8 : 1.0 [13]
Autoimmune thyroid diseases	Hashimoto's thyroiditis	7.500% [14]	7.800% [14]	Female	4.0 : 1.0 [14]
	Graves' disease	1.00%–2.00% [15]	0.750% (hyperthyroidism) [16]	Female	5.0–10.0 : 1.0 [17]
Celiac disease		1.400% [18]	1.000% [19]	Female	1.5 : 1.0 [18]
Crohn disease		0.300% [20]	0.0015%–0.331% [21]	Female	1.2: 1.0 [20]
Autoimmune atrophic gastritis		0.300%–2.700% [22]	—	Female	2.0–3.0 : 1.0 [22]
Vitiligo		0.400% [23]	0.390%–0.520% [24]	Equal	1.0: 1.0 [23]
Rheumatoid arthritis		0.380% [25]	0.200%–0.400% [25]	Female	2.5 : 1.0 [25]
Systemic lupus erythematosus		0.044% [26]	0.040% [27]	Female	8.5 : 1.0 [26]
Psoriasis		0.504% [28]	0.424%–1.884% [28]	Equal	1.0 : 1.0 [28]
Addison's disease		0.008%–0.014% [29]	0.004%–0.022% [29]	Female	1.5–3.5 : 1.0 [30]

Hashimoto's thyroiditis (HT) is the most prevalent and Addison's disease (AD) is the rarest AID. In addition, most AID exhibit gender bias toward females; notable exceptions include vitiligo and psoriasis, which affect both sexes equally, and T1D, which is more common in males. Comparing epidemiological data from the general population with prevalences from a population with T1D can reveal associations. Due to the broad variation between geographical regions [32], a comparison with German data, which is a country with comprehensively available data for numerous AID, is provided (Table 2).

**TABLE 2:** Comparison of prevalence in the general population with data from people with type 1 diabetes (T1D).

Autoimmune disease		Prevalence estimate in the German population [33]	Prevalence estimate in people with T1D in Germany [34]
Autoimmune thyroid diseases	Hashimoto's thyroiditis	2.300%	56.840%
	Graves' disease	0.412%	43.680%
Celiac disease		0.160%	15.750%
Crohn disease		0.350%	1.570%
Autoimmune atrophic gastritis		2.000% (estimate for women)	42.520%
Vitiligo		0.122%	18.110%
Rheumatoid arthritis		1.360%	5.510%
Systemic lupus erythematosus		0.046%	1.570%
Psoriasis		1.850%	9.450%
Addison's disease		0.015%	2.630%

Estimates for the German population are shown from a study that is based on billing data of statutory health insurance‐accredited physicians from the years 2012 to 2022 and included insured individuals of all ages (> 73 million people). These data are compared with findings from a longitudinal, long‐term observational study conducted at a German academic referral centre for endocrine AID, which followed 665 unselected subjects with T1D, T1D with additional AID and their first‐degree relatives between 1999 and 2020. The comparison shows that all listed AID are much more common in people with T1D than in the general population. The strongest associations with T1D according to the relative increases in prevalence are observed with autoimmune‐induced thyroid diseases (HT, Graves' disease [GD]), celiac disease (CeD), vitiligo and AD. It has also been described that in T1D, an increased presence of disease‐specific autoantibodies exists, reinforcing its link with other autoimmune disorders [34, 35]. In clinical practice, this necessitates heightened vigilance for coexisting immune‐mediated disorders in individuals with T1D. These may also include other clinically relevant conditions such as hypoparathyroidism, premature ovarian insufficiency (POI) and alopecia areata, all of which have been associated with T1D [36, 37, 38].

**FIGURE 2:**
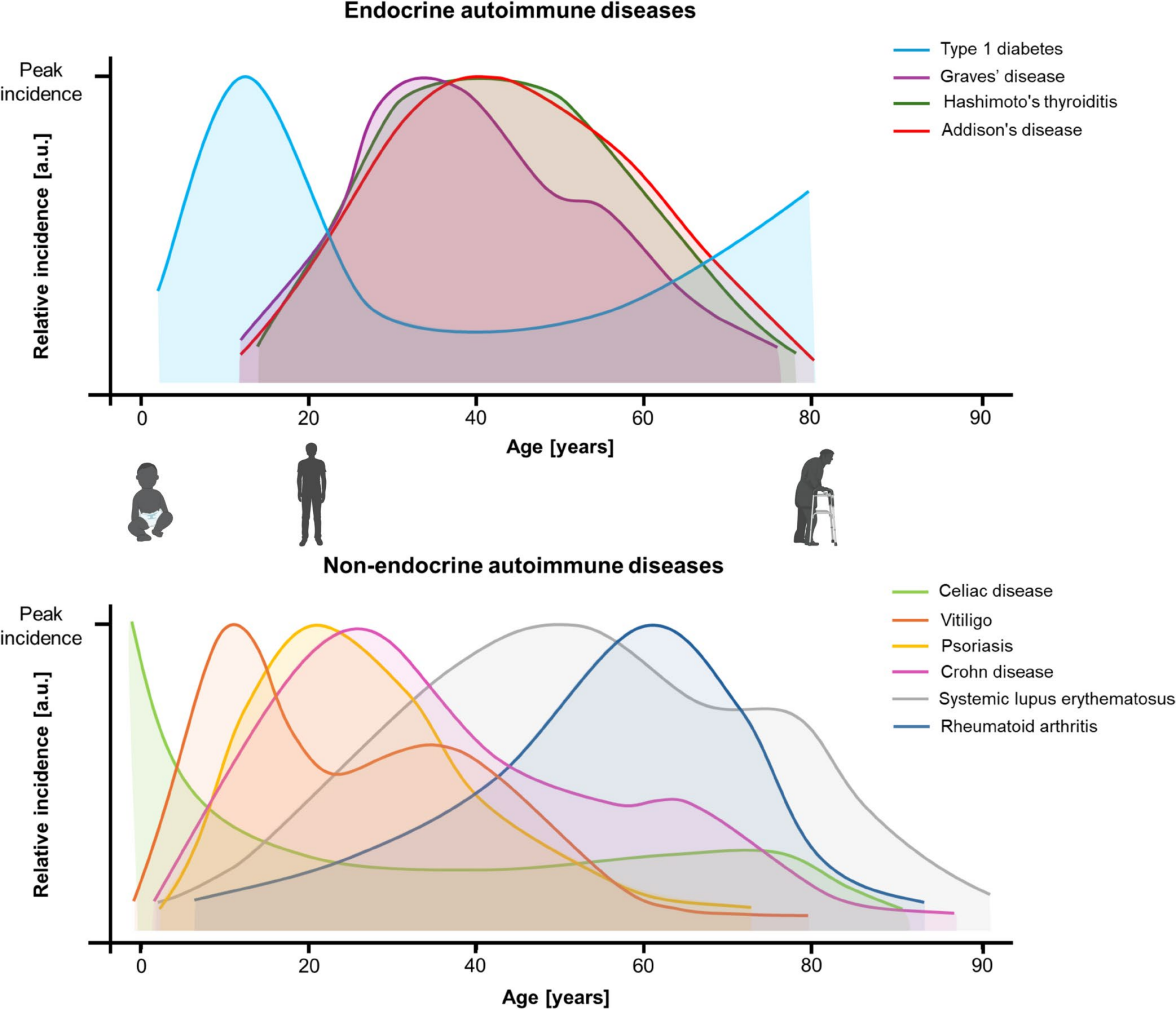
Relative incidence of autoimmune diseases (AIDs). Among endocrine AID (upper part), type 1 diabetes (T1D) typically begins in childhood or adolescence, with a lower incidence in middle‐aged adults that increases again with age. Autoimmune thyroid diseases and Addison's disease peak at a more advanced age compared to T1D. Some non‐endocrine AID (lower part) also show their peak incidence during childhood or adolescence, with celiac disease being the earliest peaking AID, even earlier than T1D. Additionally, vitiligo, psoriasis and Crohn disease are likely to appear at a young age. When interpreting the data, it is important to note that the curves do not provide information about the absolute incidence rates. The graph is based on the publications [39, 40, 41, 42, 43, 44, 45, 46, 47, 48].

It is particularly intriguing to examine the incidence rates of commonly associated AID across different age groups to elucidate their pattern of onset. The available data [39, 40, 41, 42, 43, 44, 45, 46, 47, 48] enable visualisation of the relative incidence of isolated diseases and allow for the qualitative estimation of probabilities for their early or late occurrence (Figure 2, Table S1).

Based on the incidence curves, it can be inferred that CeD, vitiligo, T1D, psoriasis and Crohn's disease are more likely to develop earlier compared to other AID. However, this does not preclude the possibility of these diseases occurring in adulthood. The prevalence of AID increases with age, peaking in middle‐aged adults (40–64 years) [49, 50, 51], indicating that age is a significant risk factor. Immune ageing plays a pivotal role in this, characterised by chronic low‐grade inflammation and failure of fundamental cellular processes in immune effector cells [52].

Regarding T1D, there are notable differences in patient characteristics between early‐onset and adult‐onset cases. Individuals with adult‐onset T1D typically exhibit a lower genetically determined risk and show a progressive loss of multiple diabetes‐associated autoantibodies over time, with glutamic acid decarboxylase (GAD) antibodies being the most persistent. Additionally, adult‐onset T1D is generally characterised by slower disease progression, as evidenced by a more gradual decline in C‐peptide levels and the continued presence of autoreactive CD8+ T lymphocytes (cytotoxic T cells) [12, 53]. In contrast, early‐onset T1D may be driven by intrinsic developmental defects of the pancreas. Metabolic differences observed in this population support the hypothesis that such primary pancreatic abnormalities could contribute to, and potentially accelerate, disease progression in early‐onset T1D compared to the adult‐onset form [53].

#### 3.1.2 Incidence of Type 1 Diabetes in Other Autoimmune Diseases

Family history of AID increases the risk of developing T1D [54, 55]. Parental CeD has been reported to result in a standardised incidence ratio (SIR, observed versus expected frequency) of 2.73 for T1D. If a parent had AD, HT, rheumatoid arthritis (RA), systemic lupus erythematosus (SLE) or GD, the SIRs were 2.41 (95% confidence interval (CI) 1.40–3.86), 2.35 (95% CI 1.78–3.06), 2.12 (95% CI 1.90–2.36), 2.04 (95% CI 1.48–2.75) and 1.86 (95% CI 1.65–2.08), respectively [56]. Among discordant AIDs in singleton siblings, AD, CeD and GD resulted in SIRs of 3.91 (95% CI 1.42–9.70), 1.92 (95% CI 1.13–3.25) and 1.83 (95% CI 1.02–3.24), respectively, reflecting significant associations with T1D risk [56]. Other studies confirm familial aggregation of discordant AIDs in parents and siblings of people with T1D [55]. One study investigating latent or subclinical autoimmunity has demonstrated that almost 60% of people with SLE or SLE first‐degree relatives show at least one autoantibody associated with another AID, and 16% (SLE first‐degree relatives) to 19% (individuals with SLE) show two or more autoantibodies. The prevalence of T1D‐specific antibodies (GAD or insulin antibodies) was ~5% in both groups. The same study has shown that additional autoantibodies were more prevalent in people with SLE and their first‐degree relatives compared to people with RA and their first‐degree relatives [57]. Overall, these results imply that the familial background of AIDs beyond T1D should be considered when evaluating the risk of T1D. Conversely, it is unsurprising that a family history of T1D increases the risk of the disease by 10‐ to 20‐fold, justifying the need for screening in this population [58].

The occurrence of at least two glandular AID in an individual is often referred to as polyglandular autoimmune syndrome (PAS) or autoimmune polyendocrinopathy. Importantly, PAS can develop sequentially over extended periods and can cluster with various non‐endocrine AID; thus, these syndromes offer an opportunity to explore the sequence in which these diseases develop. There are different subtypes of PAS (Table 3), including the rare juvenile PAS type I, which is inherited in a monogenetic manner, and the more prevalent, polygenic adult types II–IV, also including multiple gene polymorphisms [34, 59].

**TABLE 3:** Definitions and manifestations of polyglandular autoimmune syndromes (PAS) [34].

PAS subtype	Definition	Endocrine disorders/manifestations	Non‐endocrine disorders/manifestations
PAS I (juvenile type)	Coexistence of ≥ 2 glandular autoimmune diseases, typically accompanied by candidiasis Monogenetic aetiology (mutations in *AIRE* gene)	Hypoparathyroidism Hypogonadism AD/Adrenal failure	Candidiasis Moniliasis Ectodermal dystrophy Enamel hypoplasia Keratitis Hyposplenism Tubulointerstitial nephritis
PAS II (adult type)	AD and AITD and/or T1D Further endocrine and nonendocrine component diseases can be present	AD AITD T1D Hypoparathyroidism	Autoimmune gastritis Celiac disease Inflammatory bowel disease Autoimmune pancreatitis Vitiligo Alopecia Urticaria Psoriasis Rheumatoid arthritis
PAS III (adult type)	AITD and T1D (exclusion of AD) Further nonendocrine component diseases can be present	AITD T1D	Autoimmune gastritis Pernicious anaemia Celiac disease Inflammatory bowel disease Autoimmune pancreatitis Autoimmune hepatitis Primary biliary cirrhosis Vitiligo Alopecia Urticaria Psoriasis Neurodermitis Rheumatoid arthritis Systemic lupus erythematosus Myasthenia gravis Sicca/Sjögren‐syndrome
PAS IV (adult type)	Coexistence of ≥ 2 glandular autoimmune diseases not described by PAS II‐III Further nonendocrine component diseases can be present.	Hypogonadism Hypoparathyroidism Hypopituitarism AITD T1D	Autoimmune gastritis Pernicious anaemia Celiac disease Inflammatory bowel disease Autoimmune pancreatitis Primary biliary cirrhosis Vitiligo Alopecia Urticaria Pemphigus Psoriasis Neurodermitis Myasthenia gravis Sicca/Sjögren syndrome

Abbreviations: AD, Addison's disease; AITD, autoimmune thyroid disease; PAS, polyglandular autoimmune syndrome; T1D, type 1 diabetes.

An Italian survey followed 158 people with PAS I, characterised by mutations in the autoimmune regulator (*AIRE*) gene, for an average of 23.7 ± 15.1 years, with a mean age at entry of the follow‐up period of 8.3 ± 11.8 years (range 0.5–76) [60]. In two participants (1.3%), T1D was the first AID to develop, while in 11 individuals (6.9%) the disease developed subsequently. The most common initial conditions observed in this study were components of the classical triad (chronic mucocutaneous candidiasis, chronic hypoparathyroidism and AD), present in 147 (93%) individuals. Autoimmune thyroid disease (AITD) was detected in nine participants (5.7%), and autoimmune hepatitis in four participants (2.5%) at the onset of PAS I, both more frequently than T1D. AD and vitiligo had mean ages of onset of 16.3 ± 14.1 years and 17 ± 15 years, respectively, which were younger than the mean age of onset for T1D of 18.1 ± 12.6 years. The annual incidence of clinical T1D in PAS I was 1.1% [60].

A retrospective analysis of 151 individuals with PAS II in Germany reported that T1D was the most common manifestation, affecting 61%; however, it was the initial presentation in only 48% of cases [61]. Other primary manifestations included GD (19.2%), HT (17.2%), AD (14.6%) and vitiligo (12.6%) [61]. T1D and AITD showed the most frequent overlap. In people with T1D as a secondary component of PAS, the median for the interval from the index disease to T1D manifestation was ~10 years for AITD (20 cases), AD (8 cases) and vitiligo (12 cases). Whether T1D presented as the first or second manifestation of PAS did not significantly impact the interval for the onset of additional autoimmunity [61].

Further retrospective data of individuals affected by polyautoimmunity has shown for 23 affected paediatric individuals that CeD was the first autoimmune‐mediated manifestation in 39%, T1D in 26% and AITD in 22%. AITD was by far the most common secondary manifestation (78%) alongside T1D (13%), vitiligo (4%) and SLE (4%) [62]. Indeed, further evidence substantiates that children with CeD are at a significantly increased risk of subsequent T1D, with CeD‐associated antibodies frequently emerging earlier or concurrently [63, 64, 65]. A study in a Japanese population reported that 60% of subjects with GD (18 out of 30) developed GD before the onset of T1D, with most experiencing an interval of < 10 years—although some intervals approached or exceeded 20 years [66]. Furthermore, a higher prevalence of T1D‐specific autoantibodies (zinc transporter 8 (ZnT8) autoantibodies, GAD autoantibodies and insulinoma‐associated protein 2 (IA‐2) autoantibodies) was reported in individuals with AITD compared to healthy controls, also indicating an increased risk for subsequent T1D [67].

Another recent monocentric study characterised individuals with multiple AID (mean age at the onset: 23.1 ± 15.1 years) [68]. Of 111 subjects, 108 (97.3%) showed T1D, of which it occurred as the first clinical manifestation (either isolated or combined) in 87 (81%). However, in 21 individuals (19%) it was diagnosed as a secondary condition. If CeD preceded T1D (9 individuals, 43%), it took a latency of 13 years (range of 1–29 years) for T1D to develop. Further trajectories included the development of vitiligo (3 individuals, 14%), psoriasis (3 individuals, 14%), inflammatory bowel disease (2 individuals, 10%) and RA (1 individual, 5%) followed by T1D, with respective latencies of 7 years (range 2–20 years), 9 years (range 6–16 years), 19.5 years (range 19–20 years) and 22 years [68]. Interestingly, in this study, the time to develop additional autoimmunity was irrespective of the type of the first manifestation, indicating that T1D is not a specific accelerator or decelerator of autoimmune pathophysiology. This is consistent with findings from a large Swedish population‐based study demonstrating significantly increased hazard ratios for CeD (11.6; 95% CI 10.6, 12.6) and autoimmune thyroid disease (10.6; 95% CI 9.6, 11.8) among individuals with T1D [69].

A newly published retrospective, observational, matched‐cohort study evaluated the risk of and time to developing T1D in individuals with CeD, hyperthyroidism (including GD) and hypothyroidism (including HT) compared to individuals without these conditions. Using a US health claims database, the study analyzed data from 47,099 individuals with CeD, 164,830 with hyperthyroidism and 980,477 with hypothyroidism, along with matched control groups identified through propensity score matching. The mean age at index diagnosis (defined as the earliest medical diagnosis of any of the conditions studied) was 48.36 ± 21.76 years for CeD, 60.97 ± 18.23 years for hyperthyroidism and 61.93 ± 18.30 years for hypothyroidism. Results showed significantly increased adjusted hazard ratios for developing T1D in all three groups compared to matched controls: 2.54 (95% CI: 1.63, 3.97; *p* < 0.0001) for participants with CeD, 2.98 (95% CI: 2.37, 3.75; *p* < 0.0001) for participants with hyperthyroidism and 2.41 (95% CI: 2.22, 2.63; *p* < 0.0001) for individuals with hypothyroidism. These elevated risks were consistent across age groups (< 18 and ≥ 18 years) and in individuals without a family history of T1D. Furthermore, time to new‐onset T1D was significantly shorter for subjects with preexisting conditions than for those without (*p* < 0.0001) [70].

Moreover, in children with juvenile idiopathic arthritis, an adjusted hazard ratio of 1.81 (95% CI 1.03–3.17) for developing T1D compared to their healthy peers was reported [71].

### 3.2 Mechanisms Linking Type 1 Diabetes to Other Autoimmune Diseases

The removal of self‐reactive T cells within the thymus, along with the peripheral immune regulation orchestrated by regulatory T cells, is a vital process in maintaining self‐tolerance. Disruption of these mechanisms, as shown for impaired regulatory T cell function, can result in autoimmune pathologies, for example, T1D, RA and SLE [72]. Evidence suggests that a distinct cellular immune signature, characterised by elevated CD27^+^CD28^+^ CD4 T cell levels coupled with reduced CD56^dim^ natural killer cells and effector memory CD8 T cell counts, may define the immune profile of individuals with CeD, AITD and T1D [73]. Various factors contribute to the development of these cellular disturbances. The occurrence of polyautoimmunity in individuals and the familial aggregation of AID demonstrate the impact of genetic susceptibility [54]. However, the rapid increase in the incidence of, for example, T1D by 3%–4% over the past 30 years is hardly a result of very slow genetic evolution and highlights the additional role of environmental and epigenetic factors [74]. Long‐term follow‐up studies of monozygotic twins have revealed a 65% concordance rate for T1D only, hence substantiating the impact of the above factors [75, 76]. Similar concordance rates were reported for other AIDs [77, 78, 79, 80, 81].

Genetic risk factors are shared among AID [82]. Genome‐wide analyses demonstrate pervasive sharing of genetic risk loci across multiple autoimmune diseases [83, 84]. The human leukocyte antigen (HLA) region on chromosome 6, encoding cell‐surface proteins involved in antigen processing and presentation, is likely the strongest contributor to genetic susceptibility. Different mechanisms are proposed to explain this association. The molecular mimicry hypothesis suggests that external antigens resembling self‐antigens trigger autoimmunity, with some HLA variants being more efficient in presenting these. The hypothesis of a central selection failure proposes that some HLA alleles have reduced efficiency in presenting self‐peptides to mature T cells in the thymus, causing inadequate negative selection. Lastly, misfolded proteins bound to specific HLA class II molecules might become targets for autoantibodies, contributing to AID development [85, 86]. High‐risk susceptibility HLA alleles associated with multiple AID in patients with PAS type II include A1, B8, DR3, DR4, DQA1*0301, DQA1*0501, DQB1*0201 [59, 87]. In PAS III, HLA‐DRB1*03, *04, ‐DQA1*03 and ‐DQB1*02 are increased, as well as DR3‐DQB1*0201 and DR4‐DQB1*0302, with DR3 mainly conferring susceptibility for T1D [59, 87]. The DRB4*01:03:01 allele was shown to confer risk for T1D combined with CeD in children (OR of 7.84 compared to the general population) [88].

Apart from the HLA region, polymorphisms in the BTB domain and CNC homologue 2 (*BACH2*) and tumour necrosis factor alpha (*TNFα*) genes, also located on chromosome 6, seem to contribute to multiple organ autoimmunity [89, 90]. Fine‐mapping has identified two independent risk associations of T1D within the cluster of differentiation (CD)28‐cytotoxic T‐lymphocyte‐associated protein 4 (*CTLA4*) locus on chromosome 2: one near CD28, which is shared with CeD, and another intronic variant in CTLA4, shared with RA. Similarly, the *TYK2* locus on chromosome 19 displayed an independent risk association shared across SLE, inflammatory bowel disease and T1D [91]. The protein tyrosine phosphatase non‐receptor type 22 (*PTPN22*) gene on chromosome 1 was also shown to contribute to polyglandular autoimmunity [92, 93]. A list with confirmed susceptibility genes for different AIDs is given in Table 4.

**TABLE 4:** Confirmed susceptibility genes shared among different autoimmune diseases [82, 94].

Gene	Function	Mutations/Polymorphisms	Diseases
CTLA4	Encodes an immune checkpoint receptor that is expressed on T cells and inhibits T‐cell activation	C/T60 A/G49 (except for CeD)	CeD T1D HT GD PAS
PTPN22	Encodes a protein tyrosine phosphatase that strongly inhibits T‐cell activation by downregulating T‐cell receptor signalling. It is expressed in both B and T lymphocytes	+1858 C/T	T1D GD PAS
IL2‐Rα/CD25	Differentiation factor actively suppresses auto‐reactive T cells via CD25 and regulates function of natural killer cells, B cells and T_reg_	A/G*	CeD T1D GD
VDR	Expressed on immune cells and directly inhibits activated T cells. Reduces production of pro‐ inflammatory cytokines (interferon γ)	BsmI C/T Apa I A/C Taq I T/C	CeD T1D
TNF	Pro‐inflammatory cytokine.	−863 C/A −308 C/C	CeD T1D GD PAS

*Note:* * corresponds to genetic annotation, where the polymorphism inroduces a stop codon.

Abbreviations: CD, cluster of differentiation; CeD, celiac disease; CTLA4, cytotoxic T‐lymphocyte‐associated protein 4; GD, Graves' disease; HT, Hashimoto's thyroiditis; IL2‐Rα, interleukin‐2 receptor α; PAS, polyglandular autoimmune syndrome; PTPN22, protein tyrosine phosphatase non‐receptor type 22; T1D, type 1 diabetes; TNF, tumour necrosis factor; VDR, vitamin D receptor.

The contribution of multiple alleles and single nucleotide polymorphisms explains why genetic scores are utilised that integrate multiple risk loci of HLA and non‐HLA genes to identify individuals at risk [95, 96]. However, in routine practice, the American Diabetes Association (ADA) does not recommend the determination of genetic markers in people at risk of or with T1D, unless it is needed to clearly discriminate from type 2 diabetes [97].

As previously mentioned, environmental factors contribute to the initiation phase of AIDs, with many of these factors being shared across different conditions (Table 5). Viral infections and psychological stress have been implicated in the development of T1D as well as several other AID [120]. Smoking and vitamin D deficiency are also considered potential contributing factors to some AIDs discussed in this review. Moreover, epigenetic factors, such as DNA methylation, histone modifications and non‐coding RNAs, are increasingly recognised for their role in AID development. However, their complexity and the challenges associated with studying these require further investigations [76, 121, 122].

**TABLE 5:** Environmental triggers for autoimmune diseases.

Environmental risk factor	Autoimmune disease (AID)
(Viral) Infections	T1D [98], SLE [99], RA [100], AITD [101], CeD [102], psoriasis [103], Autoimmune atrophic gastritis [104], Crohn's disease [105]
(Psychological) Stress	T1D [106], SLE [107], RA [108], vitiligo [109], AITD [110], psoriasis [111]
Smoking	T1D (adult smoking only) [112], SLE [113], RA [114], psoriasis [115]
Gluten	CeD [116]
Vitamin D deficiency	T1D [117], AITD [118], SLE [119]

Abbreviations: AD, Addison's disease; AITD, autoimmune thyroid disease; CeD, celiac disease; RA, rheumatoid arthritis; SLE, systemic lupus erythematosus; T1D, type 1 diabetes.

### 3.3 Clinical‐Practical Implications

#### 3.3.1 Screening

Early detection of AID improves acute and long‐term outcomes as well as lowers healthcare costs [123, 124, 125, 126, 127]. Cost savings are driven by, for example, fewer hospitalizations and complications, while early intervention following diagnosis helps preserve overall health and quality of life [123, 124, 125, 126, 127]. T1D was shown to be the main cost driver in adult PAS, highlighting the importance of its early detection [128]. Timely diagnosis and management of T1D can reduce the incidence of diabetic ketoacidosis, improve clinical presentation at the onset of stage 3 disease, shorten hospital stays and help preserve beta cell mass—ultimately decreasing initial insulin requirements and reducing both short‐ and long‐term complications [129, 130]. Together, these factors may contribute to lowering costs.

While symptoms can provide evidence of manifest disorders, they are often relatively unspecific and may only prompt action after some therapeutic interventions have already been missed. In contrast, antibodies serve as the gold standard for early and specific indication of autoimmunity (Table 6). In individuals with pre‐existing autoimmune conditions, early detection of T1D represents a critical yet often overlooked opportunity. Therefore, antibody testing in individuals at risk, which includes people with AID(s) and eventually people with family members with AID(s), should be performed. In the context of T1D incidence among individuals with endocrine and non‐endocrine AIDs, those with AD, CeD, AITD, autoimmune atrophic gastritis, and vitiligo should be regarded as a primary focus group, as they frequently precede T1D compared to other AIDs and exhibit a high rate of coexistence with a corresponding increased prevalence. It should be noted that, whilst the predictive value of a single islet autoantibody for overt T1D over a 10‐year period is only 15%, the lifetime risk in individuals with two or more islet autoantibodies approaches 100% [8, 143]. Whereas islet cell and insulin autoantibodies are frequently present in young children developing T1D but decline with the future course, measurement of GAD antibodies in adults is preferred as serum positivity persists for years [34]. It is therefore important and recommended to test for multiple autoantibodies (e.g., insulin, GAD, IA‐2 and ZnT8) in order to improve diagnostic accuracy when screening for T1D [132].

**TABLE 6:** Essential diagnostic information pertaining to autoimmune diseases [34, 87].

Disease	Autoantibodies		Symptoms	Functional screening
	Antigen	Sensitivity, specificity [%]		
Type 1 diabetes	Glutamic acid decarboxylase (GAD)	65–75, 99	Polyuria, polydipsia, fatigue and weight loss [131]	Fasting plasma glucose (FPG) [132] Glycated haemoglobin (HbA1c) Oral glucose tolerance test (OGTT) Continuous glucose monitoring (CGM) [133]
	Islet cells (IC)	70, 99		
	Tyrosine phosphatase‐related islet antigen 2 (IA‐2)	50–90, 99		
	Pro−/insulin (I)	74, 99		
	C terminal domain of the zinc transporter 8 (ZnT8)	65–75, 99		
Hashimoto's thyroiditis	Thyroid peroxidase (TPO)	90, 99	Fatigue, weight gain, cold intolerance, constipation, depression, myalgia, menorrhagia and dry skin [134]	TSH/free thyroxine (fT4)
	Thyroglobulin (Tg)	90, 99		
Graves' disease	Thyrotropin (TSH) receptor	99, 99	Tremor, heat intolerance, weight loss, anxiety and irritability, goitre, alterations in menstrual cycles, erectile dysfunction or decreased libido, fatigue, frequent bowel movements, palpitations and others [135]	
	Thyroid peroxidase (TPO)	59–80, 99 [136, 137]		
Autoimmune atrophic gastritis	Parietal cells (PC)	90, 50	Fatigue, weakness, neurological disturbances, reflux, early satiety [22]	Endoscopy/red blood cell (RBC) count
	Intrinsic factor (IF)	80, 90		
Celiac disease	Tissue transglutaminase (TG)	90, 99	Fatigue, abdominal pain, flatulence, diarrhoea, short stature, anaemia, decreased bone density, skin changes, headaches, depression [138]	Endoscopy, biopsy
Rheumatoid arthritis	Anti‐cyclic citrullinated peptide (CCP)	20–25, 95	Musculoskeletal pain, swollen joints and stiffness [139]	Erythrocyte sedimentation rate (ESR), C‐reactive protein (CRP) and physical examination
Addison's disease	21‐hydroxylase (21‐OH) and 17 alpha hydroxylase (17‐OH)	87, 99	Hyperpigmentation, fatigue, anorexia orthostasis, nausea, muscle and joint pain, salt craving [140]	Cortisol/adrenocorticotropic hormone (ACTH)
Systemic lupus erythematosus	Antinuclear (AN)	98, 92 [141]	Fever, malaise, arthralgias, myalgia, headache, loss of appetite and weight, fatigue [142]	ESR, CRP
	Double‐stranded DNA (dsDNA)	65, 99		

The ADA recommends early and periodical screening for AITD if clinically indicated in people with T1D, as well as screening for CeD if signs or symptoms are present [144]. A report from an ADA ‘Type 1 Diabetes Screening & Awareness Roundtable’ adds to this recommendation with an expert opinion that considers screening of people with a personal or family history of AIDs for T1D as part of a stratified public health approach [145]. The European Society for the Study of Celiac Disease (ESsCD) guideline for CeD does advocate annual or biennial follow‐ups, including checks for associated autoimmune conditions, in particular AITD and T1D [146]. The American Gastroenterological Association (AGA) guideline for atrophic gastritis states that healthcare providers should have a low threshold to evaluate for T1D if the clinical picture is consistent [147].

The Italian government took a significant step forward in 2023 by passing a law that introduces a nationwide autoantibody screening program for T1D and CeD in the general population aged 1–17 years [148]. While this represents a proactive public health measure, current clinical guidelines and research initiatives continue to support targeted screening in high‐risk groups rather than population‐wide screening.

At present, the overarching goal of autoantibody testing for T1D is to prevent diabetic ketoacidosis at disease onset, enable early intervention for better clinical outcomes, and help individuals and their families prepare for clinical onset [149]. As such, it serves as a critical foundation in diabetes care. Nevertheless, respect for an individual's right to know—or not to know—their risk status remains essential [150].

Of note, artificial intelligence is being tested for its ability to predict the need for serological testing in at‐risk individuals using electronic health/medical records. In the future, it may support stratified and early screening for AID in clinical practice [151, 152].

#### 3.3.2 Disease Management

The presence of other AID in T1D is associated with therapeutic challenges, as illustrated in a retrospective study in which overall metabolic control in people with T1D with an accompanying AID was poor (mean HbA1c of 10.1% ± 1.8%) [31]. Hypothyroidism is characterised by attenuated basal plasma insulin levels, increased glucose‐induced insulin secretion, insulin resistance and weight gain [153]. As a result, HT can promote recurrent episodes of hypoglycemia and heightened glucose variability, requiring less insulin for T1D. Thyroid hormone replacement therapy can reverse these effects, thereby necessitating further adaptations to insulin dosage [154]. Moreover, in adolescents and young adults with T1D and thyroid autoimmunity, the intake of levothyroxine was found to be associated with significantly higher odds ratios (OR) for psychiatric disorders, such as depression (OR 1.63, 95% CI, 1.34–1.99), anxiety (OR 1.60, 95% CI, 1.18–2.18) and attention‐deficit/hyperactivity disorder (OR 1.71, 95% CI, 1.38–2.12) [155], which may represent a further concern. Conversely, hyperthyroidism and GD contribute to hyperglycemia through various pathways, such as heightened appetite, enhanced glucose absorption in the intestines, increased glucose production in the liver, elevated lactate output and accelerated insulin clearance and breakdown [154]. Unless thyroid dysfunction is under control with antithyroid drugs, insulin need is usually increased but needs to be closely monitored and modified once normal thyroid function has been restored [156]. Although AITD is the most frequent AID associated with T1D, its impact on T1D remains incompletely characterised. The link between the two disorders is illustrated in a study where TPO‐antibody positivity was noted in 28.8% of patients with latent autoimmune diabetes in adults (LADA). TPO‐antibody‐positive patients had higher GAD autoantibody titers, substantially lower basal C‐peptide (0.69 ± 0.16 vs. 1.9 ± 1.3 ng/mL), shorter time to insulin initiation and slightly worse glycemic control (higher HbA1c). The data support screening for thyroid autoantibodies in adults with suspected T1D because their presence signals faster progression toward insulin dependence [157].

When coexisting with untreated CeD, T1D differs from isolated T1D due to the presence of malabsorption, which may lead to an improved glycemic status but substantially elevates the risk of hypoglycemia [158]. Introduction of a gluten‐free diet normalises intestinal mucosa architecture and nutritional uptake, leading to amelioration of symptoms, albeit described effects on glucose metabolism in the literature are inconsistent [154, 156, 159]. Nevertheless, it is recommended to closely monitor insulin requirements and adjust the dosage as needed. Undeniably, the combination of these conditions substantially diminishes quality of life, as they impose strict limitations on daily activities and routines by adherence to treatment regimens [160].

Subjects with combined T1D and AD have an impaired quality of life, increased mortality and, under replacement therapies, are at increased risk of hypo−/hyperglycemia, diabetic ketoacidosis and adrenal crisis compared to patients with isolated diseases [161]. Particularly, the opposing effects of insulin and glucocorticoids make it difficult to align the treatments [161]. People with both AD and T1D undergoing glucocorticoid replacement therapy typically require a lower dose of basal insulin while needing a greater amount of prandial insulin compared to those with T1D alone [161, 162].

Although results on various micro‐ and macrovascular outcomes are heterogeneous and sometimes conflicting, an association with an increased risk for neuropathy or ischemic heart diseases in people with T1D and AID seems likely [163, 164, 165]. Interestingly, two studies have shown a benefit in terms of microalbuminuria in people with T1D and concomitant AITD [163, 165].

Beyond this, autoimmunity is also associated with POI, with unfortunate consequences for women and families with a desire to have children [68, 166, 167]. Women with POI were reported to have an odds ratio of 25.8 (95% CI 9.0, 74.1) for the prevalence of PAS prior to the index date compared to healthy controls; however, T1D was not found as a specific risk factor [168]. In people with T1D and other AIDs, it is essential to provide adequate patient education on this matter and, if needed, schedule health check‐ups with a gynaecologist [68].

## 4 Conclusions

The close link between T1D and other AID necessitates vigilance among healthcare practitioners and a coordinated approach to screening. Early detection of AID can prevent life‐threatening complications, for example, adrenal crises and/or diabetic ketoacidosis, and give those affected time to prepare for the disease and its treatment. It becomes even more important with available therapeutic interventions, such as teplizumab for T1D, which can delay clinical manifestation and the associated reduced quality of life caused by polyautoimmunity [169, 170]. Unfortunately, guidelines are often missing recommendations for screening for T1D, including dermatological guidelines for vitiligo and psoriasis, as well as rheumatological guidelines for SLE and RA [171, 172, 173, 174]. A consensus on testing for other AIDs in affected people, which harmonises recommendations across specialties, along with well‐elaborated screening protocols, would be highly beneficial. To address this gap, we have outlined important practice points (Box 1) based on this work, which may serve as a practical framework for clinicians.

BOX 1 **Important Practice Points.**

- Evaluate the personal and family history of autoimmune diseases (AID) as part of routine risk assessment.
- Autoantibody testing provides an opportunity to detect individuals at an early stage of type 1 diabetes (T1D), prior to the appearance of symptoms (for further diagnostic information see Table 5).
- Address psychological and emotional impact of early detection (e.g., anxiety) [150]. Educate and counsel to ensure informed consent prior to autoantibody testing. Clarify that the presence of ≥ 2 islet autoantibodies strongly predicts clinical T1D. Integrate psychological support into follow‐up care for individuals who test positive.
- Perform screening for multiple T1D–associated autoantibodies (glutamic acid decarboxylase [GAD], tyrosine phosphatase‐related islet antigen 2 [IA‐2], pro−/insulin and C terminal domain of the zinc transporter 8 [ZnT8]) in individuals (children, adolescents and adults) with a personal or family history of AID(s).
- Consider repeated screening for individuals with a personal or family history of AID. Children should be tested at ages 2 and 6, with age 10 recommended for adolescents [175, 176, 177].
- It is recommended that the first autoantibody‐positive test be confirmed with a second test within 3 months [178]. Presence of ≥ 2 islet autoantibodies enables the diagnosis of T1D.
- Particularly, but not solely, include GAD autoantibodies in T1D screening protocols for adults. In diagnostically uncertain cases, C‐peptide testing can help clarify the type of diabetes by assessing beta‐cell function [132].
- Conduct functional testing in individuals who test positive for autoantibodies. T1D: fasting plasma glucose (FPG), glycated haemoglobin (HbA1c), oral glucose tolerance test (OGTT) and/or continuous glucose monitoring (CGM) [178].
- Perform annual/biennial clinical checks for associated AIDs in individuals with a diagnosed autoimmune condition.
- Prompt autoantibody testing is indicated in the presence of clinical symptoms suggestive of T1D or any other AID.

On the other hand, the mechanistic similarities of AIDs provide the opportunity to expand therapies across different indications. For instance, ustekinumab, a drug used to treat psoriasis, has recently demonstrated efficacy in a preliminary study with recent‐onset T1D [179]. Moreover, rituximab, infliximab and adalimumab are used to treat different AIDs [180, 181, 182].

Overall, large prospective studies that enrol subjects with a genetic risk for PAS (before onset of AID) that include a comprehensive long‐term autoimmune follow‐up are required to gain unbiased information on the sequence of autoimmunity. The effects of regional differences on these data must also be taken into account.

T1D is often regarded as a childhood‐onset disease that plays a major role in driving multiple autoimmunity. However, two studies on polyautoimmunity show that the development of subsequent autoimmunity is independent of T1D being an index or secondary disease [61, 68]. The mean age at onset of T1D was reported to be close to the age of onset of other AIDs, such as AITD, vitiligo, AD, CeD and psoriasis [61, 68]. Consequently, no specific AID has been pinpointed as the primary driver of autoimmunity. This underscores the need for a generalised sensitivity to glandular and associated non‐endocrine polyautoimmunity. It is also important to recognise that other AID, not explicitly discussed, may coexist with T1D in clinical practice.

## Author Contributions

**Thomas Forst:** writing – review and editing. **Petra‐Maria Schumm‐Draeger:** writing – review and editing. **Matthias Schott:** writing – review and editing. **George J. Kahaly:** writing – review and editing. **Monika Kellerer:** writing – review and editing. **Stefanie Lanzinger:** writing – review and editing. **René D. Rötzer:** writing – original draft and editing.

## Conflicts of Interest

T.F. provided advisory services to Astra Zeneca, Atrogi, Bayer, Cipla, Eli Lilly, Eysense, Fortbildungskolleg, Novo Nordisk, Pfizer, Sanofi, Remynd and Roche. He also provided speaker services to Amarin, Astra Zeneca, Böhringer Ingelheim, Berlin Chemie, Cipla, Daiichi‐Sankyo, Eli Lilly, Fortbildungskolleg, MSD, Novartis, Novo Nordisk, Sanofi and Santis. M.K. provided consultancy to Abbott, AstraZeneca, Bayer GmbH, Boehringer Ingelheim, Lilly Diabetes Care, NovoNordisk and Sanofi; lecturing activities for AstraZeneca, Boehringer Ingelheim, Lilly Diabetes Care, MedLearning GmbH, Novo Nordisk, Janssen‐Cilag and Sciarc GmbH. R.D.R. is an employee of Sciarc GmbH. M.S. provided advisory services to Ipsen, Eisai, Lilly and Bayer. He also provided speaker services to Sanofi Aventis, Lilly, Diasorin, NovoNordisk and Ipsen. P.‐M.S.‐D. provided speaker services to Sanofi, Novo Nordisk, Eli Lilly, Novartis, Berlin Chemie, AstraZeneca, MSD, Boehringer Ingelheim, Hexal and Aristo Pharma. She also provided advisory services to Sanofi, Novo Nordisk, Bristol Myers Squibb, AstraZeneca, Boehringer Ingelheim, Eli Lilly and Merck. The other authors declare no conflicts of interest.

## Supporting information


**Table S1:** Extracted epidemiological data from indicated references showing incidence measures for different AIDs across the lifespan. Allocation to male and female numbers was performed where primary literature incorporated gender‐specific data. NA = no data for this time frame available.

## Data Availability

The authors have nothing to report.
